# The mediating role of job crafting in the relationship between perceived organizational support and nurses' attitudes towards artificial intelligence: a cross-sectional study based on the job demands–resources model

**DOI:** 10.3389/fmed.2026.1942864

**Published:** 2026-10-29

**Authors:** Xuan Cui, Haihong Li, Min Shi, Ting Zhao

**Affiliations:** The Affiliated Lianyungang Hospital of Xuzhou Medical University, Lianyungang, Jiangsu, China

**Keywords:** artificial intelligence, job crafting, job demands–resources model, mediation analysis, nurses, perceived organizational support

## Abstract

**Background:**

Artificial intelligence (AI) is being rapidly integrated into clinical nursing, yet many nurses remain ambivalent or resistant to these technologies. While perceived organizational support (POS) and job crafting have each been linked to nurses’ work attitudes, little is known about how they jointly shape nurses' views on AI. Drawing on the job demands–resources (JD-R) model, in which perceived organizational support represents a job resource and job crafting a proactive behaviour, this study examined whether job crafting mediates the association between POS and nurses' attitudes towards AI application.

**Methods:**

Between April and June 2026, 314 clinical nurses from a tertiary grade A hospital in China were invited via convenience sampling; 305 returned valid responses. Participants completed the Perceived Organizational Support Scale (POSS), the Nurse Job Crafting Scale (NJCS), and the Chinese version of the Attitudes towards AI Application at Work (AAAW) scale; items 14–21 of the AAAW were reverse-scored. Mediation was tested using the SPSS PROCESS macro (Model 4) with 5,000 bootstrap samples.

**Results:**

Mean item scores were 3.86 (SD = 0.80) for POS, 3.90 (SD = 0.59) for job crafting, and 3.46 (SD = 0.68) for AI attitudes. All three variables showed significant positive correlations (r = 0.502–0.715, all *p* < 0.01). Job crafting partially mediated the POS–AI attitudes relationship, accounting for 60.8% of the total effect (indirect effect = 0.248, 95% CI [0.169, 0.337]). The direct effect of POS remained significant after controlling for the mediator (b = 0.160, *p* = 0.006). Because AAAW is multidimensional, the model was also tested on theoretically distinct facets; the indirect effect was significant for positive perceptions (0.301, 95% CI [0.203, 0.403]) but not for AI-related concerns.

**Conclusions:**

Job crafting was associated with the relationship between POS and nurses’ AI attitudes in a pattern consistent with partial mediation. Organizational support and job crafting were associated with more positive attitudes toward AI, consistent with the JD-R model. Both factors may matter for AI adoption in nursing. The cross-sectional design limits causal inference, and longitudinal studies are needed to confirm these pathways.

## Background

The growing integration of artificial intelligence (AI) into healthcare is reshaping clinical nursing in ways that were hard to imagine a decade ago. AI technologies now support tasks ranging from clinical decision support and automated nursing documentation to patient risk warning, intelligent scheduling, and personalized patient education (1, 2). As the largest group of healthcare professionals delivering direct patient care, clinical nurses are both the primary end-users of these technologies and key stakeholders whose buy-in matters enormously for successful implementation (3, 4).

The evidence on nurses’ attitudes toward AI is mixed. A systematic review of 37 studies with more than 10,000 participants found guarded optimism: nurses recognized the potential of AI to improve efficiency and patient care, but also expressed concerns about ethics, algorithmic transparency, deskilling, and human-centered care (2). A qualitative systematic review of nine studies across five countries identified five recurring themes: ethical issues, increased workload from poorly designed tools, the potential for more efficient care, a belief that AI should support rather than replace nurses, and cautious optimism about AI (4). Many nurses also express concerns about job displacement, reduced clinical autonomy, and the humanistic dimensions of care (5). A recent mixed-method study of nurses in internal medicine clinics reported moderate AI anxiety and concerns about ethical violations, threats to holistic care, and patient satisfaction (6).

AI exposure and training remain limited in some settings (7), and organizational trust and leadership may also shape nurses’ responses to AI (8, 9).Perceived organizational support (POS) is the degree to which employees feel that their organization values their contributions and cares about their well-being (10). POS has drawn growing attention as a potential influence on nurses’ attitudes toward AI. Organizational support theory holds that when employees perceive organizational commitment, they develop a felt obligation to reciprocate through positive work attitudes and behaviors (10). In nursing contexts, higher POS has been consistently associated with lower burnout, reduced turnover intentions, and greater openness to workplace innovations (11, 12). A meta-analysis of 40 studies covering 23,451 nurses in 18 countries found that POS was negatively associated with turnover intention, and that cultural background, economic context, and working years moderated this association (13). Nurses who feel supported through adequate training, accessible resources, and honest communication may be more willing to approach AI with an open mind. Supporting this possibility, a study of 472 Jordanian nurses found that organizational support, together with performance expectancy and facilitating conditions, predicted continued use of electronic health records (14). However, how POS specifically relates to nurses’ attitudes toward AI remains unclear.

Job crafting refers to employees’ proactive efforts to reshape their tasks, relationships, and the meaning of work (15). Within the JD-R model, it reflects self-initiated changes that employees make to balance job demands and resources (16). In hospitals facing AI-related change, task crafting may help nurses develop AI-related skills, relational crafting may support knowledge exchange, and cognitive crafting may help nurses view AI as a complement to their expertise (15).

Previous studies have identified job crafting as a pathway linking workplace resources to outcomes such as work engagement and innovative behavior (17, 18). Among 553 Chinese clinical nurses, job crafting and thriving at work were chain mediators between POS and innovative behavior, and the total mediating effect accounted for 29.28% of the POS–innovation association (19). Among 546 newly graduated nurses, team job crafting partially mediated the association between transformational leadership and occupational well-being and explained 33.5% of the total effect (20). It is therefore plausible that POS is associated with AI attitudes not only directly but also through nurses’ self-directed job crafting. However, few studies have simultaneously examined this mediating pathway in the context of AI adoption (21–23).

The JD-R model provides a framework for these relationships (24, 25). It distinguishes job demands from job resources and proposes that resources are the main drivers of proactive work behavior (24, 25). The model has been widely used in nursing to explain how job resources translate into work-related behaviors and attitudes, including the use of new technologies (26, 27).

In this study, POS is treated as a job resource because it provides nurses with training, resources, and psychological safety to engage with AI. Job crafting is the proactive behavior through which nurses draw on that resource. The JD-R model therefore suggests a pathway in which POS is associated with job crafting, which in turn is associated with more favorable AI attitudes. To our knowledge, few studies have integrated these variables within a single JD-R account.

## Current study

This study examined whether job crafting mediates the association between POS and nurses’ attitudes toward AI. Nurses are increasingly asked to work with AI, but many remain hesitant or resistant, often because of concerns about job displacement or the dehumanization of care. POS is an established resource associated with positive work outcomes, but it seems unlikely to influence AI attitudes through a direct path alone. Drawing on the JD-R model, we propose that organizational support creates opportunities that activate nurses’ job crafting in task, relational, and cognitive domains, and that these efforts shape how nurses think and feel about AI. Few studies have simultaneously examined this mediating pathway, and the JD-R model has rarely been used to integrate these variables. This study therefore adds to the growing evidence by offering a theoretically grounded view of how organizational support may be translated into technology acceptance. The findings may help nursing leaders design more targeted strategies to improve nurses’ AI attitudes, building on both organizational support and nurses’ own initiative.

The following hypotheses were tested:

H1: POS positively predicts AI attitudes.
H2: POS positively predicts job crafting.
H3: Job crafting positively predicts AI attitudes.
H4: Job crafting mediates the relationship between POS and AI attitudes.

The hypothesized research framework is presented in Figure 1.

**Figure 1:**
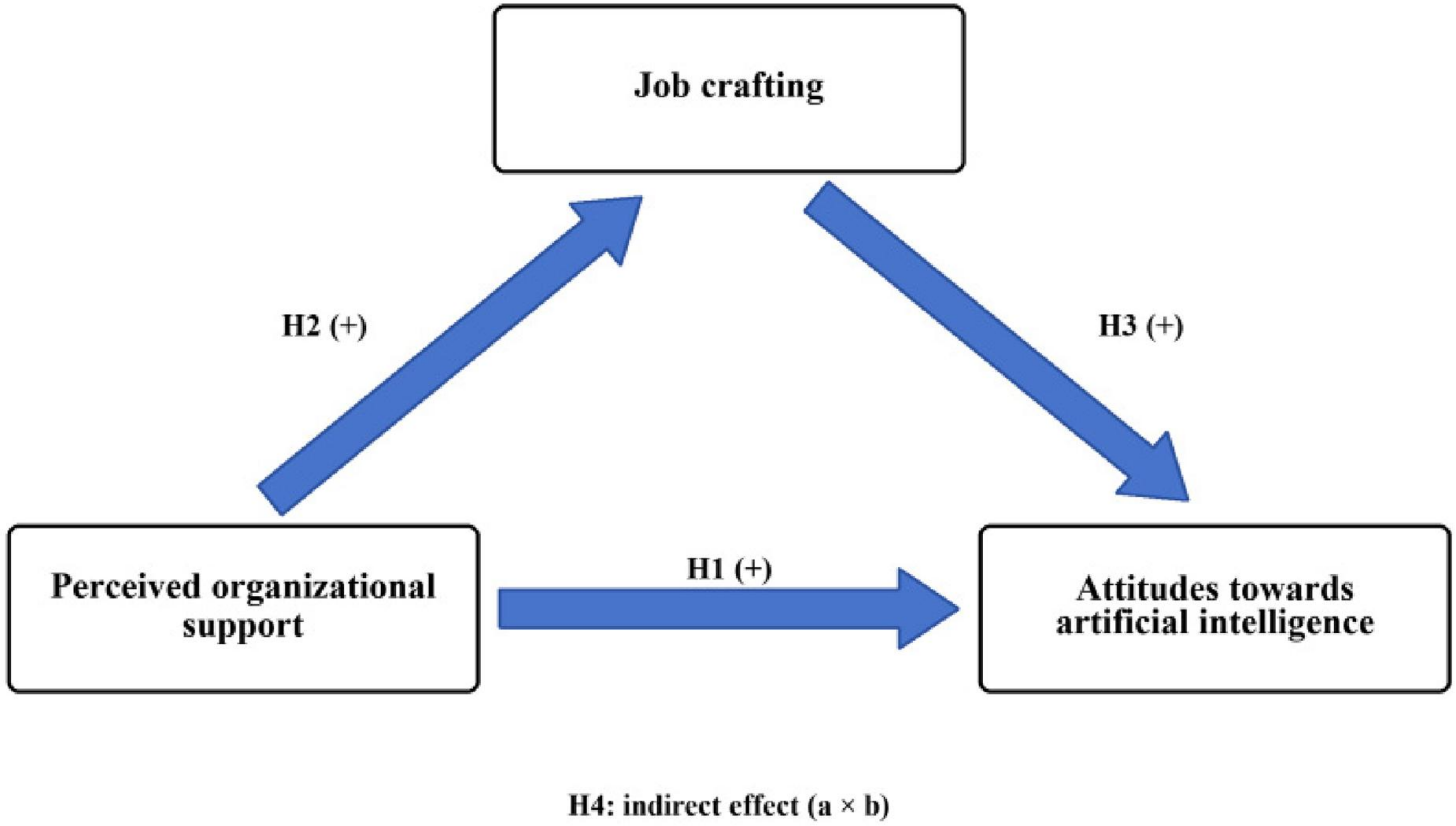
Hypothesised model based on the job demands–resources framework. POS, perceived organizational support. H1, direct association between POS and attitudes towards AI; H2, POS with job crafting; H3, job crafting with attitudes towards AI; H4, indirect effect (a × b).

## Methods

### Participants and procedures

A cross-sectional design was used. Between April and June 2026, convenience sampling was used to recruit clinical nurses from a tertiary grade A general hospital. Inclusion criteria were a valid nursing license with at least one year of clinical nursing experience and voluntary agreement to participate. Nurses on sick or maternity leave during the investigation period were excluded, as were standardized training nurses and visiting nurses. Of 314 nurses initially invited, 305 returned valid questionnaires; nine did not return a complete questionnaire, yielding a valid response rate of 97.13%.

Data were collected through the Wenjuanxing online survey platform. With support from the hospital nursing department, the questionnaire link and QR code were distributed by ward head nurses to clinical nurses. The survey included uniform instructions and an informed consent statement. To prevent duplicate responses and protect anonymity, only non-identifiable data were collected, and access was restricted to one submission per verified IP address. Because all items were required, there were no missing item-level data. All participants provided informed consent, and no incentives were offered. The final sample of 305 participants provided more than 10 observations per estimated parameter, which is consistent with common recommendations for mediation analysis.

### Measures

#### Perceived organizational support scale (POSS)

The POSS originally developed by Chen and revised by Li (28) was used to measure POS. The scale has 13 items covering two dimensions: emotional support (items 1–10) and instrumental support (items 11–13). Items are scored from 1 (strongly disagree) to 5 (strongly agree), with total scores ranging from 13 to 65; higher scores mean higher POS. There are no reverse-coded items. Cronbach's *α* in this study was 0.990.

#### Nurse job crafting scale (NJCS)

The NJCS translated into Chinese by Cao (29) was used to assess job crafting. It contains 21 items across three dimensions: cognitive crafting (7 items), relational crafting (7 items), and task crafting (7 items). Items are scored from 1 (strongly disagree) to 5 (strongly agree), with total scores ranging from 21 to 105. Higher scores indicate more job crafting behavior. There are no reverse-coded items. Cronbach's *α* was 0.969.

#### Attitudes towards AI application at work (AAAW) scale

The AAAW scale, originally developed by Park et al. (30) and translated and validated in Chinese by Li et al. (31), measures attitudes toward AI application with 25 items across six dimensions: perceived humanlikeness (4 items), perceived adaptability of AI (4 items), perceived quality of AI (5 items), AI use anxiety (4 items), job insecurity (4 items), and personal utility of AI (4 items). The original English scale was validated in three independent samples (total *N* = 2,841; CFI = 0.98, RMSEA = 0.04, SRMR = 0.04; composite reliability 0.70–0.94 across dimensions) (30). The Chinese version also showed good reliability and factorial validity (Cronbach's alpha = 0.893; CFI = 0.958, RMSEA = 0.054) (31). Items are rated from 1 (strongly disagree) to 5 (strongly agree); items 14–21 are reverse-scored according to the Chinese scoring instructions, and higher total scores reflect more positive AI attitudes. Cronbach's alpha in the present sample was 0.949.

The questionnaire also captured sociodemographic and occupational information: age, gender, educational level, marital status, years of work experience, professional title, clinical department, position type, interest in AI, prior AI training, and prior experience using AI software.

Ethics StatementThis study was conducted in accordance with the Declaration of Helsinki. Ethical approval was obtained from the Institutional Review Board of a tertiary hospital in China (Approval No.: LW-20260402001-01). All participants were fully informed about the study's purpose and procedures, and they provided informed consent online before completing the questionnaires. The survey was anonymous and confidential, and participation was entirely voluntary. No incentives were offered for participation.

## Statistical analysis

Data analysis proceeded in four stages. First, descriptive statistics and Pearson correlations were computed. Second, mediation was tested using the SPSS PROCESS macro (Model 4) with 5,000 bootstrap samples to estimate 95% confidence intervals for the indirect effect. Covariates (educational level, position type, AI interest, AI training, and AI use experience) were prespecified on theoretical and clinical grounds and were included in all models. Third, the measurement model was examined by confirmatory factor analysis (CFA), from which average variance extracted (AVE), composite reliability (CR), the Fornell–Larcker criterion, and the heterotrait–monotrait ratio (HTMT) were computed. Finally, a sensitivity analysis without covariates was performed, and secondary mediation analyses were conducted for two theoretically distinct AAAW facets: positive/functional perceptions and AI-related concerns.

Descriptive statistics including mean, standard deviation, skewness, and kurtosis were calculated before the main analyses. Harman's single-factor test was used to assess common method bias because all data were self-reported (32). Several procedural remedies were applied during data collection to mitigate common method bias: questionnaires were completed anonymously and voluntarily, respondents were assured that there were no right or wrong answers, and validated scales with clear instructions were used.

## Results

All three core variables showed acceptable normality: skewness values ranged from −0.09 to 0.26 and kurtosis from −0.70 to 1.43, all within the |2| cutoff (Tabachnick & Fidell, 2013). Internal consistency was acceptable across all scales. Participant demographic characteristics are shown in Table 1. Descriptive statistics and bivariate correlations for the study variables are presented in Table 2.

**Table 1:** Sample characteristics (*N* = 305).

Characteristic	Category	n (%)
Gender	Female	280 (91.8)
	Male	25 (8.2)
Age (years)	18–<25	32 (10.5)
	25–<30	69 (22.6)
	30–<40	135 (44.3)
	40–<50	47 (15.4)
	50–60	22 (7.2)
Education	Diploma	36 (11.8)
	Bachelor	255 (83.6)
	Master	13 (4.3)
	Doctorate	1 (0.3)
Marital status	Married	210 (68.9)
	Unmarried	90 (29.5)
	Other	5 (1.6)
Professional title	Nurse	34 (11.1)
	Senior nurse	84 (27.5)
	Supervisor nurse	144 (47.2)
	Associate chief nurse	38 (12.5)
	Chief nurse	5 (1.6)
Work experience (years)	<1	11 (3.6)
	1–<2	14 (4.6)
	2–<5	45 (14.8)
	5–<10	61 (20.0)
	10–<20	119 (39.0)
	≥20	55 (18.0)
Position type	Clinical nursing	277 (90.8)
	Nursing management	25 (8.2)
	Other	3 (1.0)
Interest in AI	Yes	267 (87.5)
	No	38 (12.5)
AI devices available	Yes	140 (45.9)
	No	165 (54.1)
Prior AI training	Yes	74 (24.3)
	No	231 (75.7)
Prior AI software use	Yes	195 (63.9)
	No	110 (36.1)

**Table 2:** Descriptive statistics and correlation matrix.

Measure	POS	Job Crafting	AI Attitudes
POS	—	.715**	.502**
Job Crafting	.715**	—	.560**
AI Attitudes	.502**	.560**	—
Mean	3.86	3.90	3.46
SD	0.80	0.59	0.68
Skewness	−0.09	0.00	0.26
Kurtosis	−0.44	−0.70	1.43

** p *<* *0.01.*

Mediation Analysis.

Before estimating the structural model, we examined the measurement model using confirmatory factor analysis with dimension parcels (two for POS, three for job crafting). The two-factor model showed acceptable fit and was superior to the one-factor model (*Δχ*^2^(1) = 489.0, *Δ*CFI = 0.279). AVE and composite reliability were adequate, and the Fornell–Larcker criterion and HTMT supported discriminant validity. Because AAAW is multidimensional, theoretically distinct facets were analyzed in addition to the total score (Table 4).

Table 3 and Figure 2 show the results. POS significantly predicted job crafting (path a: b = 0.508, SE = 0.031, t = 16.43, *p* < 0.001, 95% CI [0.421, 0.596]); together with the covariates, the model explained 54.0% of the variance in job crafting. Job crafting significantly predicted AI attitudes (path b: b = 0.488, SE = 0.080, t = 6.13, *p* < 0.001, 95% CI [0.351, 0.626]). The total effect of POS on AI attitudes was significant (path c: b = 0.408, SE = 0.045, t = 9.06, *p* < 0.001). After controlling for job crafting, the direct effect remained significant (path c′: b = 0.160, SE = 0.059, t = 2.73, *p* = 0.006), consistent with partial mediation. The indirect effect via job crafting was 0.248 (boot SE = 0.043, 95% CI [0.169, 0.337]); because the bootstrap confidence interval excluded zero, the indirect effect was statistically significant, accounting for 60.8% of the total effect. POS, job crafting, and the covariates together explained 34.5% of the variance in AI attitudes (R^2^ = 0.345). A sensitivity analysis without covariates showed a similar pattern.

**Table 3:** Path coefficients, covariate estimates and indirect effect (PROCESS model 4).

Path/parameter	b	SE	t	*p*	95% CI
a: POS → Job crafting	0.508	0.031	16.43	<0.001	[0.421, 0.596]
b: Job crafting → AI attitudes	0.488	0.080	6.13	<0.001	[0.351, 0.626]
c: POS → AI attitudes (total)	0.408	0.045	9.06	<0.001	—
c′: POS → AI attitudes (direct)	0.160	0.059	2.73	0.006	—
Indirect effect (a × b)	0.248	0.043	—	—	[0.169, 0.337]
Covariates → Job crafting					
Education (bachelor or above)	0.064	0.073	0.87	0.385	—
Position (clinical nursing)	−0.101	0.083	−1.22	0.223	—
AI interest	0.224	0.072	3.08	0.002	—
AI training	0.057	0.061	0.94	0.348	—
AI use experience	0.049	0.052	0.94	0.348	—
Covariates → AI attitudes					
Education (bachelor or above)	−0.053	0.083	−0.63	0.529	—
Position (clinical nursing)	−0.126	0.094	−1.33	0.184	—
AI interest	0.183	0.084	2.18	0.030	—
AI training	−0.013	0.069	−0.19	0.850	—
AI use experience	0.146	0.059	2.48	0.014	—
R^2^ (job crafting model)	0.540				
R^2^ (AI attitudes model)	0.345				

SE, standard error; Coeff, unstandardized coefficient; X, predictor variable; M, mediator variable; Y, outcome variable. Covariates in the model included educational level, position type, AI interest, AI training, and AI use experience; their coefficients are reported in the lower panels. b, unstandardized coefficient; CI, confidence interval.

**Figure 2:**
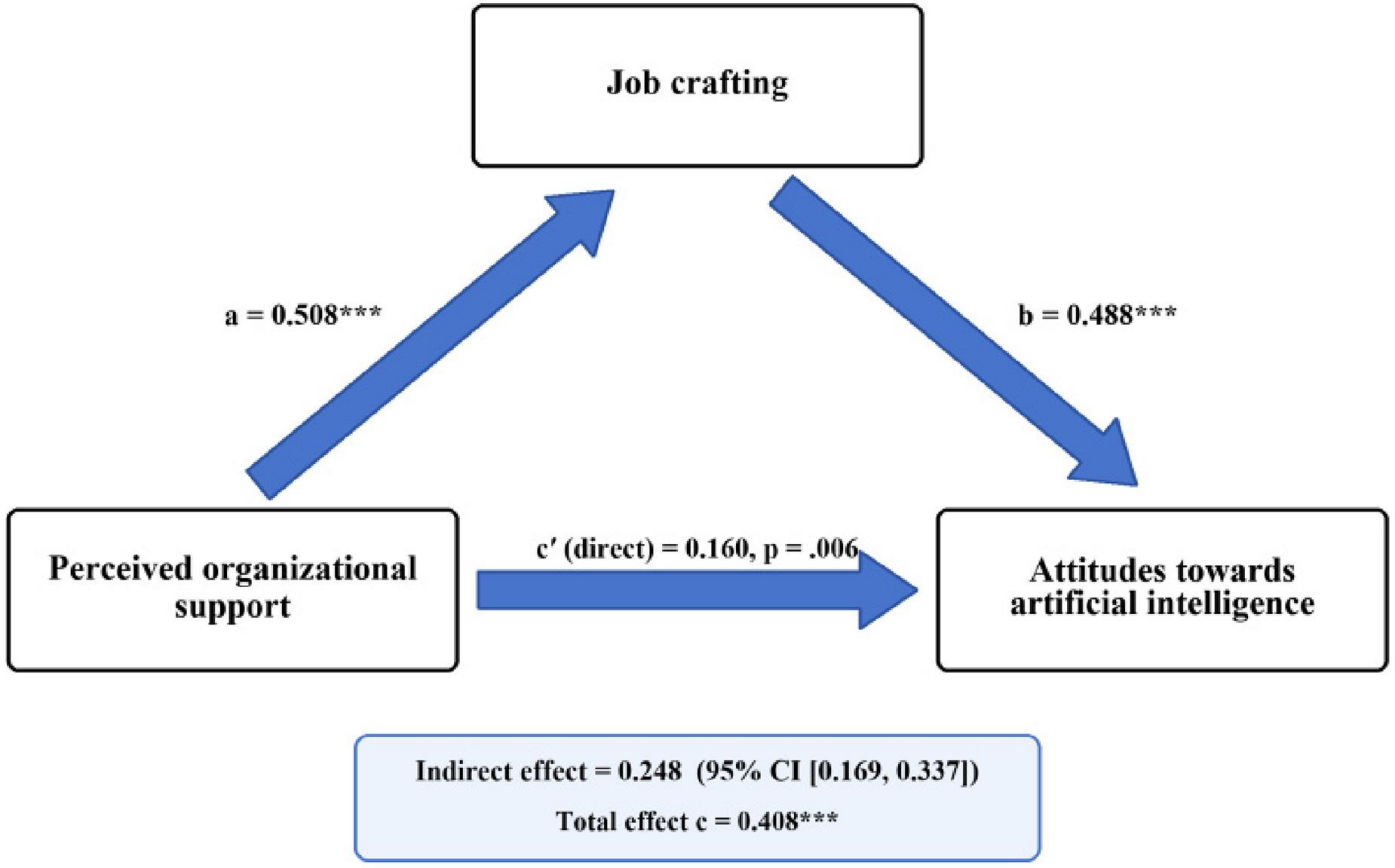
Path coefficients of the mediation model. Values are unstandardized coefficients; the dashed line indicates the direct effect. Covariates (educational level, position type, AI interest, AI training and AI use experience) were included in all models. ***p < .001.

### Facet-level analyses

Because AAAW is multidimensional, we conducted secondary mediation analyses for two theoretically distinct facets (Table 4). The indirect effect was significant for the positive-perception facet, which comprised perceived humanlikeness, perceived adaptability, perceived quality, and personal utility (17 items), but not for the concerns facet, which comprised AI use anxiety and job insecurity (8 items).

**Table 4:** Facet-level mediation results (POS → job crafting → AAAW facets).

Outcome facet (no. of items)	b (JC → facet)	Indirect effect	95% CI	% of total effect
Positive perceptions (17)	0.593	0.301	[0.203, 0.403]	58.9
Concerns: AI use anxiety + job insecurity (8)	−0.266	−0.135	[−0.314, 0.005]	—

Higher scores on the concerns facet indicate greater AI use anxiety and job insecurity.

## Discussion

This study examined whether job crafting was associated with the relationship between POS and nurses’ attitudes, using the JD-R model as a guiding framework. In this single-center sample of 305 clinical nurses from one tertiary hospital in China, all four hypotheses were supported. The indirect pathway through job crafting was sizeable: approximately three-fifths (60.8%) of the total effect of POS on AI attitudes was accounted for by job crafting.

Consistent with H1, POS was positively associated with nurses’ attitudes. This finding aligns with earlier work showing that organizational support is associated with openness toward workplace innovations among healthcare professionals (11, 12, 14). The total effect (b = 0.408) suggests that nurses who feel that their hospital values their contributions and well-being are more likely to hold favorable views of AI. Within the JD-R model, organizational support is a job resource that enables engagement with new technology (24). When hospitals invest in training and resources and communicate clearly about why AI is being introduced, nurses may view AI as a quality improvement effort rather than a cost-cutting measure that threatens their professional autonomy (26, 27). The direct effect of POS on AI attitudes remained significant after job crafting was included in the model (b = 0.160, *p* = 0.006). POS may therefore operate through more than one channel, some mediated by job crafting and others not. The unmediated portion may reflect the felt obligation and trust described in organizational support theory, which can feed directly into positive work attitudes (10).

Several recent studies have examined other mechanisms underlying nurses’ AI attitudes in Chinese nursing samples. Liu et al. (2025) found that AI anxiety mediated the relationship between AI literacy and nurses’ attitudes toward AI (33). Chen et al. (2025) reported that technology trust and perceived value mediated the association between eHealth literacy and attitudes toward AI use in nursing (21). Xu et al. (2026) found that hospital hierarchy was associated with AI attitudes through AI literacy and anxiety (22), and Yu et al. (2026) reported that AI literacy mediated the association between change fatigue and AI attitudes among county-level nurses (23). These studies focus mainly on literacy-related and affective mechanisms. The present study contributes by examining an organizational resource and a proactive behavioral mechanism within the JD-R framework. Because the design is cross-sectional, temporal ordering cannot be established. Job crafting accounted for 60.8% of the total effect of POS on AI attitudes. This is a substantial share, which indicates that nurses’ own proactive efforts may play an important role in translating organizational resources into positive technology attitudes. Within the JD-R model, job crafting acts as a bridge between POS as a job resource (materials and psychological safety) and nurses’ feelings about AI (24). The three dimensions of job crafting may contribute to this process in different but complementary ways. Task crafting helps nurses acquire AI-related skills on their own initiative and may strengthen their perceived ability to work with AI (24). Relational crafting builds informal peer networks in which AI knowledge is shared outside formal training channels (15). Cognitive crafting allows nurses to reframe the meaning of AI for their work and to view it as something that can support rather than threaten their clinical judgment (15, 16). Together, these dimensions may create a self-reinforcing cycle of growing competence and confidence. This interpretation is consistent with evidence that job crafting can act as a psychological resource supporting work engagement and adaptability during organizational change (17–20).

Our finding that job crafting was associated with the POS–AI attitude link differs from Ekinci Demirelli (2025), who found that cognitive and relational job crafting did not significantly mediate the POS–job performance relationship (34). This difference suggests that the mediating role of job crafting may depend on the outcome examined. The indirect effect in our study was sizeable and statistically significant. However, the proportion mediated depends on the magnitude of the total effect and on the outcome and model examined, so it should not be directly compared with proportions reported in studies using different outcomes or models (19, 20). AI attitudes may be particularly responsive to self-initiated learning and cognitive reframing, which are central to job crafting (15). One statistical consideration is that POS was a strong predictor of job crafting (b = 0.508, R^2^ = 0.540), which is consistent with the JD-R model's claim that job resources are primary drivers of proactive work behavior (16, 35). The confirmatory factor analyses indicated that the two constructs were empirically distinguishable, but their strong association suggests that discriminant validity should be re-examined in larger samples.

This study adds to the evidence by testing a mediation pathway connecting organizational support to nurses’ AI attitudes within the JD-R model. The use of bootstrapped confidence intervals avoids reliance on normality assumptions for the indirect effect (36). Several limitations should be considered when interpreting these results. The cross-sectional design is the most important limitation. Although the hypothesized direction of effects is theoretically grounded, reverse or reciprocal relationships cannot be ruled out. For example, nurses with more positive AI attitudes may be more likely to perceive organizational support and to engage in job crafting. Recent longitudinal work suggests that job crafting behaviors and well-being outcomes may have reciprocal relationships over time (37). Longitudinal and experimental designs are needed before causal claims can be made, and mediation effects estimated from cross-sectional data can be biased relative to their longitudinal counterparts (38). The sample was drawn from a single tertiary hospital in one Chinese province through convenience sampling, which limits generalizability to nurses in other hospitals, regions, or healthcare systems. The high response rate (97.13%) may partly reflect the local organizational culture or social desirability. A recent multi-center study across Beijing, Sichuan, and Yunnan (*N* = 878) found regional variation in nurses’ AI literacy and attitudes (7), suggesting that findings from a single hospital in Jiangsu may not represent the broader Chinese nursing population. Multicenter studies with geographically diverse samples, including hospitals of different tiers and regions, would be valuable.

All measures were self-reported, so common method variance cannot be fully excluded; procedural remedies were applied, and future studies should use multi-source or temporally separated measures (32). Two further limitations should be acknowledged. The POSS showed high internal consistency, suggesting possible item redundancy; future studies may use more differentiated measures of organizational support. Second, because AAAW is multidimensional, the six dimensions were analyzed as theoretically distinct facets in addition to the total score; the mediating pathway was evident for positive perceptions but not for AI-related concerns. Future studies using more differentiated measures of organizational support and behavioral rather than attitudinal outcomes would help establish the stability of these findings.

These findings have implications for nursing leaders in similar hospital settings. Because POS was associated with AI attitudes both directly and through job crafting, hospital administrators may benefit from building supportive organizational climates during AI implementation. Concrete steps include transparent communication about why AI is being adopted and what it means for nurses’ work, protected time for AI-related training, and visible recognition of nurses’ efforts to adapt to technological change (10). Research suggests that nurse managers are often excluded from decisions about technology design and implementation, which can contribute to resistance and burnout; engaging them early in the AI integration process is therefore important (39). Job crafting accounted for approximately three-fifths (60.8%) of the total effect, which points to a practical direction: interventions that strengthen nurses’ proactive behaviors may help foster positive AI attitudes. Nurse managers could implement structured job crafting interventions that encourage nurses to (a) seek AI-related learning opportunities and adjust their task boundaries (task crafting), (b) build interdisciplinary networks for AI knowledge exchange (relational crafting), and (c) reframe AI as a complement to, rather than a replacement for, professional nursing judgment (cognitive crafting) (15, 16). These interventions are consistent with the JD-R model's emphasis on strengthening job resources and mobilizing them through proactive behavior ^(^24). Different nurses will need different kinds of support. Previous research has shown that job crafting tendencies vary as a function of personality traits and career orientation (40). Nurses with lower baseline job crafting tendencies may benefit from structured mentorship that scaffolds proactive behaviors, while those already strong in job crafting might be ready for advanced AI training and champion roles in AI implementation teams. This differentiated approach echoes calls in the nursing management literature for personalized support strategies during technological transformation (39).

Longitudinal designs that track nurses through actual AI rollouts would be a logical next step, allowing researchers to determine whether changes in POS and job crafting precede changes in AI attitudes over time. Such designs could also test reciprocal effects, which have been documented in the job crafting literature (37). Multilevel studies examining hospital-level factors (organizational AI readiness, nursing leadership, and unit climate) and individual-level mediators would add contextual understanding (39). Intervention trials testing whether job crafting training changes nurses’ AI attitudes and, downstream, their actual AI adoption behavior would provide the strongest test of the proposed pathways (24). Qualitative and mixed-methods research could complement these efforts by capturing nurses’ experiences as they craft their jobs in response to AI-related changes. Finally, cross-cultural comparative studies, such as comparisons between Chinese nurses and nurses in other healthcare systems, could help identify which findings are context-specific and which may be more universal (34). More broadly, future work should move beyond conventional predictor–mediator–outcome models and examine for whom and under what conditions job crafting matters, how AI-specific crafting behaviors unfold over time, and how nurses’ attitudes evolve during real-world AI implementation (41).

## Conclusions

This study provides evidence that job crafting was associated with the relationship between POS and nurses’ attitudes toward AI application, consistent with partial mediation. Within the JD-R model, organizational support may operate as a job resource associated with nurses’ job crafting, which in turn was associated with more favorable AI attitudes. The indirect pathway accounted for approximately three-fifths (60.8%) of the total effect, suggesting that nurses’ own proactive efforts may play an important role in translating organizational resources into positive technology attitudes. Facet-level analyses showed that this indirect association was evident for positive perceptions but not for AI-related concerns. These findings suggest that supportive organizational environments and structured job crafting interventions may support AI adoption in nursing. Because the study was cross-sectional, these associations should not be interpreted causally; longitudinal and intervention studies are needed to confirm the proposed pathways.

## Data Availability

The raw data supporting the conclusions of this article will be made available by the authors, without undue reservation.
